# Sizing the lung of mechanically ventilated patients

**DOI:** 10.1186/cc10034

**Published:** 2011-02-14

**Authors:** Jennifer S Mattingley, Steven R Holets, Richard A Oeckler, Randolph W Stroetz, Curtis F Buck, Rolf D Hubmayr

**Affiliations:** 1 Division of Pulmonary and Critical Care Medicine, Mayo Clinic College of Medicine, 200 First Street SW, Rochester, MN 55905, USA; 2 Division of Respiratory Therapy, Mayo Clinic College of Medicine, 200 First Street SW, Rochester, MN 55905, USA

## Abstract

**Introduction:**

This small observational study was motivated by our belief that scaling the tidal volume in mechanically ventilated patients to the size of the injured lung is safer and more 'physiologic' than scaling it to predicted body weight, i.e. its size before it was injured. We defined Total Lung Capacity (TLC) as the thoracic gas volume at an airway pressure of 40 cm H2O and tested if TLC could be inferred from the volume of gas that enters the lungs during a brief 'recruitment' maneuver.

**Methods:**

Lung volume at relaxed end expiration (Vrel) as well as inspiratory capacity (IC), defined as the volume of gas that enters the lung during a 5 second inflation to 40 cm H2O, were measured in 14 patients with respiratory failure. TLC was defined as the sum of IC and Vrel. The dependence of IC and Vrel on body mass index (BMI), respiratory system elastance and plateau airway pressure was assessed.

**Results:**

TLC was reduced to 59 ± 23% of that predicted. Vrel/TLC, which averaged 0.45 ± 0.11, was no different than the 0.47 ± 0.04 predicted during health in the supine posture. The greater than expected variability in observed Vrel/TLC was largely accounted for by BMI. Vrel and IC were correlated (r = 0.76). Taking BMI into account strengthened the correlation (r = 0.92).

**Conclusions:**

We conclude that body mass is a powerful determinant of lung volume and plateau airway pressure. Effective lung size can be easily estimated from a recruitment maneuver derived inspiratory capacity measurement and body mass index.

## Introduction

The low tidal volume trial of the ARDS Network (the ARMA trial), supported by a long list of preclinical and clinical studies, has unequivocally established that mechanical ventilation with large tidal volumes (VTs) can be injurious to the lungs of patients with acute lung injury (ALI) or the acute respiratory distress syndrome (ARDS) [1]. However, neither ARMA nor subsequent clinical trials resolved questions and controversies about 'best PEEP [positive end-expiratory pressure]' management, about the efficacy of recruitment maneuvers, or about the efficacy of specific modes of ventilation or, most importantly, how to best tailor ventilator mode and settings, including VT, to the needs of individual patients. ARMA established that a VT of 6 mL/kg of predicted body weight (PBW) was safer than one of 12 mL/kg PBW and was associated with a survival benefit. Since the main determinants of PBW and those of the size of the normal lung are the same (namely, height and gender [2,3]), the ARMA protocol, in effect, targeted VT to the size of the lung before it was injured. Because it is widely acknowledged that the size of the recruitable lung (Gattinoni's 'baby lung') is decreased in ALI [4] and because that decrease was undoubtedly nonuniform across ARMA patients, it is probable that, in both trial arms, patients with severe disease were ventilated with VTs that were disproportionately larger than those patients with mild disease. Indeed, this argument was put forth recently by Chiumello and colleagues [5], who measured the functional residual capacity of the lungs of patients with ALI. The ARMA protocol did provide a mechanism for lowering VT to 4 mL/kg PBW in patients in whom plateau airway pressure (Pplat) would have otherwise exceeded 30 cm H_2_O. However, the use of this threshold as a surrogate for severe lung impairment has yet to be validated and is obviously influenced by the choices of PEEP, VT, respiratory muscle activity, and the mechanical properties of the chest wall [6]. Indeed, esophageal manometry-based estimates of intrathoracic pressure in recumbent patients with ALI or ARDS suggest that the recoil properties of the chest wall may in fact dominate Pplat [7,8].

This small observational study on 14 mechanically ventilated patients was motivated by our belief that scaling VT to the size of the injured lung is safer and more 'physiologic' than scaling it to PBW (that is, to its size before it was injured). Considering this premise, we set out to measure the total lung capacity (TLC) of 14 mechanically ventilated patients with respiratory failure and to test whether measuring the volume of gas that enters the lungs during a brief inflation to 40 cm H_2_O is sufficient to predict TLC at the bedside. We show that there is a reasonable correlation between the inflation maneuver-derived inspiratory capacity (IC) and the thoracic gas volume (TGV) at relaxed end-expiration (Vrel) and that, in the supine posture, Vrel/TLC is determined in large part by the body mass index (BMI). We also confirm earlier reports that suggested great variability in parenchymal deformation of patients with injured lungs when VT is targeted to PBW as opposed to effective lung size [5] and address the feasibility and challenges of making IC measurements by means of commercially available mechanical ventilators.

## Materials and methods

### Patient population

Fourteen hemodynamically stable (mean arterial pressure of greater than 60 mm Hg, no inotrope support) patients, who were mechanically ventilated with a fractional inspired oxygen (FiO_2_) concentration of not more than 0.65 and who were sufficiently sedated to tolerate a 5-second lung inflation to an airway pressure of 40 cm H_2_O without inducing respiratory effort, were studied. The protocol was approved by the Institutional Review Board, and informed consent was obtained from each patient's legally authorized representative.

### Experimental interventions

Patients were mechanically ventilated with an Engstrom GE Carestation ventilator (GE Healthcare, Madison, WI, USA) at settings previously determined by the primary care providers (Table 1). The GE Carestation ventilator provides a means to estimate TGV based on nitrogen dilution [9] with a ± 10% confidence (according to the manufacturer's specifications). The pressure and flow sensors of a NICO cardiopulmonary monitor (Philips Respironics, Wallingford, CT, USA) were placed in line between the endotracheal tube and the Y-connector of the ventilator tubing. PEEP was set to 0 cm H_2_O (initial 4 patients) or 5 cm H_2_O (subsequent 10 patients) and TGV at relaxed end-expiration (Vrel) was measured 5 minutes later. Data from the 4 patients, in whom Vrel was estimated at zero end-expiratory pressure (ZEEP), are identified as such throughout this report. The ventilator was then switched to a pressure control mode at a rate of three breaths per minute so that the lungs could be inflated to an airway pressure of 40 cm H_2_O for 5 seconds. Inflation and deflation volume, flow, and pressure were recorded using the NICO monitoring module. IC, defined as the amount of gas entering the lungs between the pressures of 0 or 5 and 40 cm H_2_O was recorded on the NICO system, so it could be subsequently compared with the volume estimates derived from the ventilator's digital display. IC measurements were made in triplicate, whereby maneuvers with phasic respiratory muscle activity as judged by pressure and flow patterns were rejected *post hoc *from further analysis. The inflation maneuver was to be aborted on the basis of predefined safety termination criteria but in no instance were these met (mean blood pressure of less than 55 mm Hg or a 20% change from baseline; heart rate of less than 60 or greater than 140; oxygen desaturation of less than 85%; and distress). The experiment concluded with a repeat measurement of Vrel before the patients were returned to their original ventilator settings.

**Table 1:** Baseline characteristics and ventilator settings

			Mechanical ventilation					
							
Patient	Age, years	Sex	Indication	Time, hours	**BMI, kg/m**^**2**^	**PEEP, cm H**_**2**_**O**	P/F, mm Hg	VT, mL
1	37	M	Acute lung injury	229	71	10	270	550
2	37	F	Influenza pneumonia	108	39	10	262	300
3	55	M	Acute histoplasmosis	44	26	10	265	570
4	59	M	Encephalopathy	19	24	5	453	450
5	70	F	Health care-associated pneumonia	108	35	8	108	380
6	29	F	Encephalopathy	135	21	5	436	350
7	73	F	Health care-associated pneumonia	92	29	5	171	400
8	79	M	Airway protection	34	30	8	165	420
9	66	F	Health care-associated pneumonia	35	29	5	290	340
10	53	F	ARDS-sepsis	62	38	10	240	370
11	60	M	Ventilator-associated pneumonia	11	37	7.5	121	500
12	74	M	Health care-associated pneumonia	15	21	5	272	450
13	79	M	Sepsis, myocardial infarction	22	35	5	385	550
14	59	F	Community-acquired pneumonia	21	42	10	216	385

ARDS, acute respiratory distress syndrome; BMI, body mass index; F, female; M, male; PEEP, positive end-expiratory pressure; P/F, partial pressure of oxygen to fractional concentration of inspired oxygen ratio; VT, tidal volume.

### Analyses and statistical methods

Normal values for TLC, vital capacity (VC), and residual volume were derived from reference values provided by Goldman and Becklake [10]. The elastance of the respiratory system (ERS) was derived from PEEP, Pplat, and VT at baseline ventilator settings. To account for the recumbent posture, the predicted normal values for VC were reduced by 5% and subdivided so that predicted Vrel and IC came to occupy 13% and 87% VC, respectively [11]. Data were graphed and analyzed with Excel 2003 (Microsoft Corporation, Redmond, WA, USA) and JMP 8 (SAS Institute Inc., Cary, NC, USA). Unless specified, all data are presented as mean ± standard deviation. Correlations between variables were assessed by linear regression. Statistical significance was accepted at a *P *value of less than 0.05.

## Results

### Patient demographics

Clinical diagnosis and baseline ventilator data were obtained from the patients' electronic medical records (Table 1). Eleven of 14 patients had an inflammatory or infectious lung insult often manifest as ALI. The remaining 3 patients were encephalopathic, had varying degrees of dependent atelectases, and had been intubated largely for airway protection. All had been mechanically ventilated at PEEP and VT settings consistent with ARDS Network recommendations [1]. As a group, the patients were overweight, two individuals having a BMI of greater than 40 kg/m^2^.

### Lung volumes and their subdivisions

As expected, TLC was substantially reduced in the majority of patients, averaging 59% ± 23% of the predicted value (Table 2). The reduction in TLC was a result of a proportional decrease in Vrel and IC, which averaged 58% ± 23% and 61% ± 26% of normal, respectively. Since we consider TLC to be the best estimate of effective lung size and hence of the degree of lung impairment, we examined its relationship to ERS and Pplat. While there was a statistically significant correlation between Pplat and TLC (*r *= -0.66), the relationship was dominated by two outliers (patients with preserved, that is, normal TLC). Consequently, neither Pplat nor ERS helped predict the reduction in effective lung size in patients with lung injury.

**Table 2:** Respiratory system volumes and pressures

Patient	TLC, liters	TLC, percentage of predicted	Vrel, liters	IC, liters	IC-ICex, mL	**Pplat, cm H**_**2**_**O**	**ERS, cm H**_**2**_**O/liter**
1	2.30	0.35	0.77	1.52	48	29	51
2	2.31	0.50	1.18	1.13	78	25	79
3	6.47	1.01	3.32	3.14	126	11	18
4	4.63	0.60	2.54	2.09	^a^	12	24
5	2.38	0.44	1.11	1.27	^a^	19	29
6	4.05	0.79	2.15	1.90	84	14	26
7	2.26	0.54	1.14	1.11	106	30	63
8	3.77	0.62	1.87	1.90	40	20	29
9	1.67	0.43	0.69	0.99	77	22	50
10	1.53	0.29	0.33	1.20	169	18	22
11	3.51	0.55	1.48	2.03	220	21	27
12	3.52	0.54	2.15	1.36	125	18	29
13	6.23	1.08	2.52	3.71	340	12	13
14	3.23	0.57	1.06	2.17	210	20	26
Mean ± SD	3.42 ± 1.54	0.59 ± 0.23	1.59 ± 0.85	1.82 ± 0.80	135 ± 0.87	19 ± 6	35 ± 19

^a^Missing paired inflation and exhaled volume data. ERS, elastance of the relaxed respiratory system; IC, inspiratory capacity; ICex, exhaled volume from total lung capacity; Pplat, plateau airway pressure; SD, standard deviation; TLC, total lung capacity; Vrel, lung volume at relaxed end-expiration.

**Figure 1:**
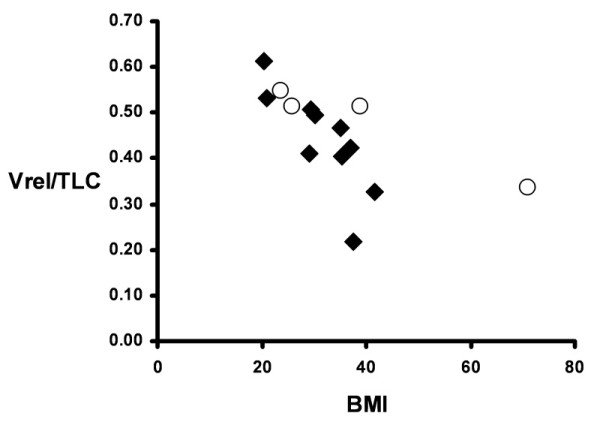
**Relationship between lung volume at relaxed end-expiration (Vrel) expressed as a fraction of total lung capacity (TLC) and body mass index (BMI)**. Open symbols identify measurements of patients 1 to 4, in whom Vrel was measured at zero end-expiratory pressure. Except for the outlier with a BMI of 71, in the expected population BMI range, Vrel/TLC declines by 1% TLC for each 1 kg/m^2 ^increase in BMI (*r *= -0.81).

For the group, the ratio of Vrel/TLC, which averaged 0.45 ± 0.11, was not statistically different than the 0.47 ± 0.04 predicted for these individuals during health in the supine posture [11]. However, the greater-than-normal variability in observed Vrel/TLC was accounted for largely by BMI (*r *= -0.63) (Figure 1). In contrast, neither ERS nor Pplat measured at baseline ventilator settings was a meaningful predictor of the variability in Vrel/TLC (*r *= 0.18 and -0.11, respectively).

**Figure 2:**
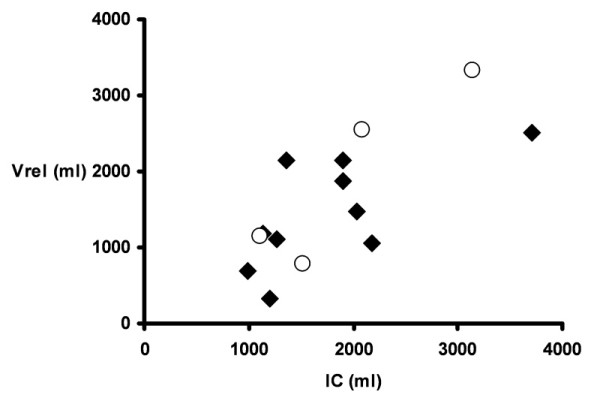
**Relationship between relaxation volume, lung volume at relaxed end-expiration (Vrel), and inspiratory capacity (IC)**. Open symbols identify measurements of patients 1 to 4, in whom Vrel was measured at zero end-expiratory pressure. The remaining Vrel measurements were made at a positive end-expiratory pressure of 5 cm H_2_O.

With the exception of patients 3 and 13, who essentially had normal lung volumes, IC was reduced, averaging 61% ± 26% of the predicted normal value for the entire group. Inspiratory flow invariably fell to zero during the 5-second inflation to 40 cm H_2_O, consistent with previous observations on the time course of recruitment of atelectatic regions in anesthetized humans [12]. The volume of expelled gas during the subsequent passive exhalation to Vrel was smaller than IC in all instances. The difference between IC and expelled gas volume averaged 8% ± 4% IC, reflecting stress relaxation and subsequent derecruitment of lung units. The ratio of IC/TLC, which averaged 0.55 ± 0.11, was no different than would have been predicted for normal lungs in this patient sample (0.53 ± 0.04). It follows that Vrel and IC were strongly correlated (*r *= 0.76) (Figure 2). Adding BMI to this model further increased the strength of the correlation (*r *= 0.92), so that TLC could have been estimated from BMI and IC within ± 0.4 L in all but two instances.

### Disease-related variability in lung size and ventilator management

**Figure 3:**
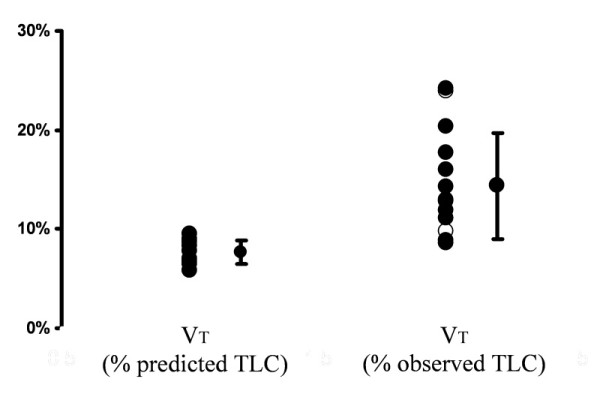
**Distribution of tidal volumes (VTs) expressed as a percentage of predicted total lung capacity (TLC) (left) and as a percentage of observed TLC (right)**. Open symbols identify measurements of patients 1 to 4, in whom lung volume at relaxed end-expiration was measured at zero end-expiratory pressure.

Since providers had scaled VT to PBW, the variability in VT when expressed as a percentage of predicted TLC was relatively small (Figure 3). For the group, VT averaged 6.8 ± 1.0 mL/kg PBW, which corresponded to 7.6% ± 1.2% of the predicted TLC. However, when VT is expressed a percentage of the observed TLC, it becomes apparent that VT occupied between 9% and 24% of the patients' lungs' capacity. For a person with normal lungs, this amounts to breathing with a VT of between 0.51 and 1.59 L. It should be noted that Pplat was less than 30 cm H_2_O in each instance, indicating that a Pplat threshold of 30 cm H_2_O does not guard against hyperventilation of aerated, recruitable regions of the injured lung.

### Feasibility and bias of inspiratory capacity measurements using commercial mechanical ventilators

Because the GE Carestation ventilator, which was used in these experiments, does not provide a numeric display of delivered volume when set in a pressure control mode, we compared ventilator-recorded expired volumes following TLC inflations with those measured with NICO. On average, the expired volume displayed on the ventilator was 5% ± 10% smaller than that measured with NICO. In part, this discrepancy reflects *post hoc *adjustments of ventilator-displayed volumes to account for temperature, humidity, and tubing compliance. As recently reported, precision, accuracy, and handling of volume information differ widely among commercially available mechanical ventilators [13].

## Discussion

The main conclusion from this small observational study is that measuring the IC of intubated patients helps predict effective lung size. Our premise entering this study was that sizing the recruitable lung is important for individualizing patient care. Our research did not test the imperative of this premise. Nevertheless, we find its underlying rationale compelling. It is generally accepted that lungs, particularly when injured, are vulnerable to additional damage by both cyclic recruitment/derecruitment and overinflation. The two injury mechanisms frequently coexist in the same lung. While prevention of the former calls for an increase in parenchymal stress (usually in the form of PEEP), prevention of the latter mandates a stress reduction, which is usually accomplished by limiting Pplat. With increasing lung impairment, the upper and lower volumes and hence stress safety boundaries within which both imperatives may be accomplished approach one another. In other words, the 'safe' inflation pressure amplitude, defined as the difference between optimal PEEP (one that maximizes recruitment) and a 'safe' Plat (one that minimizes the risk of overdistension), approaches zero or may even assume a negative value. Whereas sizing the recruitable lung does not address the choice of best PEEP or mean airway pressure per se, it does provide information about the probability that a chosen VT will encroach on upper or lower lung volume (or both) or stress safety boundaries.

We assumed that the TGV at a transrespiratory system pressure (PRS) of 40 cm H_2_O provides a reasonable estimate of the injured lungs' total capacity. In normal humans, TLC is almost completely determined by the size and recoil properties of the lungs because the lungs' compliance near TLC approaches zero whereas that of the chest wall remains finite. As a result, in upright normal humans, the intrathoracic pressure near TLC approximates 10 cm H_2_O [14]. The widely accepted plateau pressure threshold of 30 cm H_2_O as a surrogate of stress injury risk is implicitly based on these estimates. It is now apparent that the lungs of many recumbent patients, particularly those with increased BMI or distended abdomens or both, are not fully expanded at a PRS of 30 cm H_2_O [6]. Therefore, we defined TLC as the TGV at a PRS of 40 cm H_2_O. It is nevertheless likely that, in patients with extensive alveolar flooding and collapse or with morbid obesity or with both, even a PRS of 40 cm H_2_O does not guarantee full lung inflation. The choice of 40 cm H_2_O thus represents a compromise between patient safety and biologic certainty.

Our data are entirely in line with observations by Chiumello and colleagues [5], who emphasized the large between-patient variability in lung strain when VT is scaled to PBW. Since Chiumello and colleagues defined strain as the fractional volume change between Vrel and the lung volume at end-inflation, it may be assumed that patients with the smallest Vrel, those with the largest PBW, and those who were ventilated with high levels of PEEP generated the largest strain estimates. In contrast, TLC and IC were not measured directly or reported, so that lung deformation relative to lung capacity (that is, VT/TLC) cannot be inferred from the data of Chiumello and colleagues [5]. We favor VT/TLC as a surrogate of the deformation experienced by aerated alveoli. In a normal lung, alveolar size is uniform at TLC, so that regional VT/TLC may be treated as an index of regional alveolar ventilation [15]. Since in patients with ARDS the mechanical properties of aerated alveoli were found to be relatively normal [5], our reasoning applies to injured lungs as well.

We set out to measure Vrel and consequently IC at/from a volume at ZEEP. We abandoned this approach after four patients because reducing airway pressure to ZEEP frequently induced coughing, always runs the risk of oxygen desaturation, and was not essential for the objectives of our experiment. While the small sample size precludes a statistical evaluation of this change in experimental design, we are unable to detect the expected bias (lower Vrel/TLC and greater IC when Vrel is measured at ZEEP) in our data. Over 50% of inflations to 40 cm H_2_O yielded an acceptable IC estimate, even though we refrained from using neuromuscular blocking agents. Repeat IC estimates (available in 10 of 14 patients) varied by less than 12%, averaging ± 5% for the group. None of our attempts to inflate the thorax to 40 cm H_2_O pressure had to be aborted for cardiovascular reasons. Limiting the duration of inflation to 5 seconds undoubtedly enhanced the tolerance of the IC 'recruitment' maneuver. It is of note that, within the limits of our flow detection capabilities (>1 L/minute), a 5-second inflation appeared sufficient to fully expand all recruitable lung units. This observation is in keeping with computer tomography-based estimates of alveolar recruitment of atelectatic lung regions [12].

While we expected that Vrel and, by inference, IC would serve as surrogates of lung impairment, namely of disease-related loss of lung units, we were surprised how strongly Vrel/TLC correlated with BMI. This observation underscores the importance of chest wall mechanics on lung function of recumbent patients with injured lungs. It is very much in line with recent esophageal manometry-based estimates of chest wall recoil in this population and undermines the rationale for limiting airway inflation pressure and, by inference, PEEP therapy to a singular Pplat value [8,16]. On a related note, we note that lung injury had little effect on the expected relationships between Vrel, IC, and TLC. This implies that mass loading of the lung by chest wall and abdomen more or less offsets the anticipated effects of dependent 'lung collapse' on Vrel of aerated units and that the potential for lung recruitment in our small patient sample was modest [17,18]. In this context, it should be noted that the elastance of the chest wall in contrast to chest wall recoil pressure may well have been normal. As previously reported in obese volunteers with normal lungs, abdominal distension is expected to cause a rightward shift of the chest wall pressure volume curve without necessarily altering its shape [19].

Measuring the IC by means of the inherent hardware/software systems of commercially available mechanical ventilators can be challenging. Bench tests of mechanical ventilators used in our practice generally support the manufacturer's stated volume accuracy of ± 10% (data not shown). Compensation algorithms accounting for tubing compliance, gas temperature, and humidity vary greatly among vendors [13]. Therefore, we caution against an uncritical acceptance of exhaled volume displays when estimating IC or TLC in intubated, mechanically ventilated patients.

## Conclusions

We have provided evidence that measuring the volume of gas that enters the lungs during a brief inflation to 40 cm H_2_O, when adjusted for body weight/habitus, is sufficient to estimate the capacity of the injured lung at the bedside. We did not and cannot offer an opinion on the critical size of any IC- or TLC-based VT scaling factor nor do we know of specific data on its interactions with mean lung volume or PEEP. Consistent with hypotheses put forth by Chiumello and colleagues [5], we believe that many prior studies on the topic of ventilator-associated lung injury, including those dealing with best PEEP, were confounded by variability in VT/TLC and related lung injury mechanisms. Eliminating this variability in future studies might be a step forward.

The dependence of Vrel on BMI, which we have observed, indirectly supports the esophageal manometry-based conclusions of Talmor and colleagues [8] and those of Loring and Weiss [16] and thereby undermines reliance on a uniform plateau pressure target. While keeping Pplat below 30 cm H_2_O remains a reasonable initial care goal, we draw attention to the importance of BMI as a determinant of Vrel/TLC and will be less hesitant to exceed this threshold in patients with abdominal distension, but preserved TLC. Alternatively, we are likely to reduce VT to less than 6 mL/kg PBW long before Pplat reaches 30 cm H_2_O in nonobese patients with small effective lung capacities. Needless to say, validation of these approaches will require preclinical and clinical efficacy trials.

## Key messages

• Total lung capacity (TLC), defined as thoracic gas volume (TGV) at an airway pressure of 40 cm H_2_O, is reduced to varying degrees in mechanically ventilated patients with injured lungs.

• TLC can be calculated by measuring the TGV at relaxed end-expiration (Vrel) and then adding the inspiratory capacity (IC), defined as the volume of gas which enters the lungs during a 5-second inflation to an airway pressure of 40 cm H_2_O.

• Because in recumbent patients body mass and habitus are important determinants of Vrel, TLC may be estimated with reasonable accuracy from IC and body mass index alone.

• Future clinical trials in patients with injured lungs should consider data on chest wall mechanics and effective lung capacity.

## Abbreviations

ALI: acute lung injury; ARDS: acute respiratory distress syndrome; BMI: body mass index; ERS: elastance of the respiratory system; IC: inspiratory capacity; PBW: predicted body weight; PEEP: positive end-expiratory pressure; Pplat: plateau airway pressure; PRS: transrespiratory system pressure; TGV: thoracic gas volume; TLC: total lung capacity; VC: vital capacity; Vrel: lung volume at relaxed end-expiration; VT: tidal volume; ZEEP: zero end-expiratory pressure.

## Competing interests

The authors declare that they have no competing interests.

## Authors' contributions

JSM and SRH screened and identified patients, obtained informed written consent, carried out all bedside measurements, and contributed to the data analysis. RAO contributed to study design and participated in study conduct and data analysis. RWS and CFB participated in study conduct and, together with SRH, were responsible for validating methods and approach at the bench. RDH conceived the study, participated in its design and coordination, and helped to draft the manuscript. All authors read and approved the final manuscript.
